# *Rosa roxburghii* tratt residue as an alternative feed for improving growth, blood metabolites, rumen fermentation, and slaughter performance in Hu sheep

**DOI:** 10.3389/fvets.2024.1397051

**Published:** 2024-06-19

**Authors:** Huijie Li, Xinyu Song, Wenxuan Wu, Chuanshe Zhou

**Affiliations:** ^1^Key Lab of Animal Genetics, Breeding and Reproduction in the Plateau Mountainous Region, Ministry of Education, College of Animal Science, Guizhou University, Guiyang, China; ^2^Institute of New Rural Development, Guizhou University, Guiyang, China; ^3^Key Lab of Agro-Ecological Processes in Subtropical Region, Institute of Subtropical Agriculture, Chinese Academy of Sciences, Changsha, China

**Keywords:** *Rosa roxburghii* Tratt residue, growth, blood metabolites, rumen fermentation, Hu sheep

## Abstract

The utilization of agro-industrial by-products, such as fruit residues, presents a promising strategy for providing alternative feed to ruminants amidst rising prices and limited availability of traditional roughage. In this study, we investigated the effects of *Rosa roxburghii* tratt residue, a local fruit residue in Guizhou province of China, on the growth, blood metabolites, rumen fermentation, and slaughter performance of Hu sheep. Ninety-six sheep were randomly divided into four groups, namely control, treatment 1, treatment 2, and treatment 3, and fed diets containing 0, 10, 20, and 30% *Rosa roxburghii* Tratt residue, respectively. Feeding varying levels of *Rosa roxburghii* Tratt residue showed no significant differences in dry matter intake, average daily gain, or the ratio of dry matter intake to average daily gain. However, sheep in the group fed with 30% *Rosa roxburghii* Tratt residue showed the highest gross profit. Plasma albumin content was lower in groups fed with *Rosa roxburghii* Tratt residue-containing diets compared to the control group (*p* < 0.05). Additionally, diet treatment 3 decreased plasma creatinine levels compared to control and treatment 1 (*p* < 0.05). Sheep in treatment 2 and treatment 3 exhibited higher plasma high-density lipoprotein level than control and treatment 1 (*p* < 0.05), as well as increased total cholesterol levels compared to control (*p* < 0.05). There were no significant differences in other plasma metabolites. Rumen pH, N-NH_3_, volatile fatty acids, and methane levels did not differ significantly among the four groups. However, feeding diets treatment 2 and treatment 3 resulted in decreased water holding capacity and increased shear force compared to control and treatment 1 (*p* < 0.05). Furthermore, pH, red chromaticity (a*), yellowness index (b*), and luminance (L*) were unaffected among the four groups of sheep. In conclusion, the inclusion of up to 30% *Rosa roxburghii* Tratt residue had no adverse effects on growth performance, allowing for feed cost savings without impacting rumen fermentation parameters. *Rosa roxburghii* tratt residue also showed benefits in improving plasma protein efficiency and enhancing lipid metabolism, albeit with limited effects on meat quality. Considering its affordability, *Rosa roxburghii* Tratt residue presents a practical choice for low-cost diets, ensuring economic returns.

## 1 Introduction

To meet the increasing demand for cattle and sheep, exploring alternatives to traditional roughage is crucial. Utilizing agro-industrial by-products, particularly fruit residues, holds promise in this regard. Fruit residue, a by-product of fruit processing or crushing, offers advantages such as high production levels, low cost, and moderate nutrient content, making it a viable ingredient for ruminants (1). Several studies have demonstrated its potential as a substitute for conventional feed, showing no adverse effects on growth performance. For instance, Massod et al. (2) fed crossbred calves varying amounts of apple residue as a substitute for corn, observing no significant differences in growth performance or nutrient digestibility. Similarly, Sharif et al. (3) found that feeding orange pulp to lambs did not affect their feed intake or growth performance negatively. Molosse et al. (4) reported that grape residues could enhance the growth rate of lambs. Another study by Hao et al. (5) found that adding sea-buckthorn residue increased metabolizable protein supply and the average daily gain of sheep, although it was advisable to limit the addition level to 16% to avoid negative impacts on nutrient digestion. These findings collectively support the effective utilization of fruit residue by ruminants, offering a practical and scientifically backed approach to alternative feeding strategies.

*Rosa roxburghii* Tratt is a perennial wild deciduous shrub with a slightly sour taste, astringent flavor, strong aroma, and crisp texture (6). Guizhou Province is the pioneering region in cultivating, developing, and utilizing RRT resources, making it the sole province in China and globally to engage in large-scale planting and industrial development (7). RRT earned its reputation as the ‘king of VC’ due to its exceptionally high concentration of Vitamin C in its juice. Each 100 grams of RRT juice contains up to 3,500 mg of Vitamin C, making it 500 times higher than apple and pear juice, 100 times higher than citrus juice, and 40 times higher than lemon juice (8). Moreover, RRT also contains *Rosa roxburghii* Tratt polysaccharide, which possesses various beneficial functions such as antioxidation, immune enhancement, anti-coagulation, anti-tumor (9) properties, blood glucose reduction, and improvement of anti-oxidative stress ability in mice (10). *Rosa roxburghii* Tratt extract demonstrates effectiveness in human medicine by reducing blood sugar (11) and blood lipid levels (12), inhibiting tumor development (13), and protecting liver function (14).

*Rosa roxburghii* Tratt residue is the by-product of fresh *Rosa roxburghii* Tratt physical juicing, mainly generated over a 45-day period from mid-August to late September. *Rosa roxburghii* Tratt residue exhibits high moisture content ranging from 62.1 to 69.5%, rendering it susceptible to decay and contributing to resource wastage and environmental pollution. Regrettably, a significant quantity of *Rosa roxburghii* Tratt residue has been indiscriminately discarded during *Rosa roxburghii* Tratt production seasons until now. Hence, there is an imperative to explore practical means of utilizing *Rosa roxburghii* Tratt residue.

We hypothesized that *Rosa roxburghii* Tratt residue may be fed to meat sheep without having an adverse effect on their growth and metabolism because of its comparatively rich nutritional composition. In order to investigate the effects of varying quantities of *Rosa roxburghii* Tratt residue on the growth performance, blood metabolites, rumen fermentation, and slaughter performance of Hu sheep, we tested our hypothesis by providing meat sheep feed containing *Rosa roxburghii* Tratt residue. The findings of this study aim to provide a theoretical foundation for the scientific utilization of *Rosa roxburghii* Tratt residue in ruminants.

## 2 Materials and methods

### 2.1 Animal care

The present experiment received approval from the Animal Ethics Committee of Guizhou University (EAE-GZU-2023-E042).

### 2.2 Experimental design and animal management

The *Rosa roxburghii* Tratt residue was obtained from a large-scale *Rosa roxburghii* Tratt juice processing factory in Longli county, Guizhou province, China. The proximate chemical compositions of *Rosa roxburghii* Tratt residue were determined as follows: dry matter 31.50%, gross energy 18.19 MJ/kg dry matter, crude protein 11.99% dry matter, ether extract 1.43% dry matter, neutral detergent fiber 53.71% dry matter, acid detergent fiber 47.22% dry matter, and organic matter 97.03% dry matter, respectively.

Ninety-six male finishing twins of Hu sheep, averaging 60 (±1.4) days in age and 24.22 (±1.26) kg in body weight (mean ± standard difference), were randomly divided into four blocks: control, treatment 1, treatment 2, and treatment 3. Each block consisted of three replicates with eight individual sheep in each replicate. Animals were provided one of four diets differing in *Rosa roxburghii* Tratt residue level. The control sheep were fed a basal diet without *Rosa roxburghii* Tratt residue, while treatment 1, treatment 2, and treatment 3 sheep received diets containing 10, 20, and 30% *Rosa roxburghii* Tratt residue on a dry matter basis, respectively. *Rosa roxburghii* Tratt residue replaced the corresponding level of peanut vine (on a dry matter basis) for treatment 1, treatment 2, and treatment 3. The consumed diet by sheep was in the form of pelleted total mixture ration. The ingredients and chemical composition of the diet fed to the sheep are presented in Table 1.

**Table 1:** Ingredients and chemical composition of diets fed to sheep (air-dried basis).

Item	Control	Treatment 1	Treatment 2	Treatment 3
Ingredients				
Stevia residue	27	27	27	27
Peanut vine hay powder	30	20	10	
*Rosa roxburghii* Tratt residue		10	20	30
Peanut hull powder	3	3	3	3
Corn	27	27	27	27
Soybean meal	2	2	2	2
Soybean hull	1	1	1	1
Wheat bran	2.5	2.5	2.5	2.5
Palm oil granules	5.1	5.1	5.1	5.1
NaCl	0.5	0.5	0.5	0.5
CaHPO_4_	0.9	0.9	0.9	0.9
Premix^1^	1	1	1	1
Total	100	100	100	100
Chemical composition				
Dry matter	90.4	90.1	90.5	90.3
Metabolizable energy	9.1	9.2	9.2	9.2
Crude protein	11.9	12.0	12.1	12.02
Organic matter	90.9	91.2	91.4	91.5
Neutral detergent fiber	48.0	48.3	48.6	48.9
Acid detergent fiber	34.5	35.0	35.9	35.1

One kilogram of premix contained Fe 100.0 mg, Cu 17.0 mg, Mn 72.5 mg, Zn 122.0 mg, Co 0.50 mg, Se 0.30 mg, I 0.80 mg, V_A_ 13000 IU, V_E_ 68.5 mg, V_D_ 700 IU.

Before the trial, the sheep were dewormed to ensure body hygiene and underwent a 7-day adaptation period for regrouping. Animals were then subjected to a 10-day pre-feeding period followed by a 35-day trial duration. The diet was formulated according to the nutrient requirements of meat-type sheep and goats (15). Sheep were fed *ad libitum* twice a day at 08:00 and 17:00, and had free access to exercise and water throughout the study.

### 2.3 Dietary composition

Approximately 200 g of dietary sample from each block was collected daily and pooled at the end of the trial. The composited dietary sample was continuously dried in a blast oven at 65°C for 48 h. After drying, the samples were ground and crushed using a 40-mesh screen. The gross energy of the sample was determined using an oxygen bomb calorimeter (6,400 CALORIMETER, Parr). The dry matter (method 934.01), crude protein (method 968.06), and ash (method 942.05) content were determined following AOAC methods (16). The neutral detergent fiber and acid detergent fiber were determined using the FIBRETHERM automatic system FT12 (Germany) according to the method of Van Soest et al. (17). The organic matter content was calculated using the following formula: organic matter (%) = 100% − ash (%).

### 2.4 Growth performance and economic benefits

On the 1st and 36th days of the trial period, all sheep were weighed before the morning feeding. These weights were recorded as the initial body weight and the final weight, respectively. The provided feed and leftover were carefully documented throughout the experiment. From these data, the dry matter intake, average daily gain, average net gain, and the ratio of dry matter intake to average daily gain were calculated.

The cost of artificial and veterinary drugs was not considered in the analysis because the experimental sheep were large-scale farmed and received the same feeding management as that for ordinary Hu sheep, except for different diets. Therefore, the weight gain profit and cost were calculated using the following formula, which was based on the feed cost, feed intake, and weight gain data of the Hu sheep during the experiment.

Feed cost (CNY/sheep) = [dry matter intake (g/sheep/d)/1,000] × test days(d) × feed price (CNY/kg).

Weight gain income (CNY/sheep) = (final weight − initial weight) × live sheep price (CNY/kg).

Gross profit (CNY/sheep) = Weight gain income − Feed cost.

### 2.5 Plasma biochemical metabolites

In each replicate, four sheep (the selecting criteria in one block is 3 sheep for the biggest body weight, 6 sheep for the medium body weight, and 3 sheep for the least body weight) were selected for blood sample Research Topic at the end of the experiment. Ten mL of blood was drawn from the jugular vein using a disposable heparin sodium anticoagulant vacuum. The blood samples were then centrifuged at 805 × g for 15 min and divided into 3-mL centrifuge tubes. Plasma metabolites, including total protein, albumin, alkaline phosphatase, alanine transaminase, aspartate transaminase, creatinine, high-density lipoprotein, total cholesterol, triglyceride, and urea, were measured using a fully automated biochemical analyzer (BS-360VET, Mindray).

### 2.6 Rumen fermentation

On day 36 after blood sampling, two sheep were randomly selected from each pen (totally 6 sheep per block) to obtain rumen fluid before morning feeding. A gastric tube ruminal fluid sampler was orally inserted into the rumen to collect the fluid. The pH was immediately measured using a portable pH meter (PHS-3C). The remaining ruminal fluid was filtered using four layers of gauze and then centrifuged at 1425 × g for 10 min. The supernatant was collected for N-NH_3_ and volatile fatty acids analysis. VFA concentration was analyzed using a gas chromatograph (GC-2010-plus, Shimadzu, Japan) equipped with an SH-Rtx-WAX column (30.00 m × 0.25 mm × 0.25 μm). The flame hydrogen ion detector (FID) and gasification chamber were set at temperatures of 220°C and 200°C, respectively. The initial column temperature was set at 100°C and increased to 150°C at a rate of 5°C per minute. Crotonic acid was used as the internal standard (18).

### 2.7 Slaughter performance

Six sheep closest to the average body weight in each block were slaughtered to collect longissimus dorsi for later analysis. The sheep were fasted for 24 h and water for 12 h before being weighed, and this weight was recorded as their pre-slaughter live weight. After evisceration, the carcass weight was measured. The carcass percentage was calculated using the formula: carcass weight divided by pre-slaughter live weight. The pH_45 min_ and pH_24 h_, water holding capacity, shear force, and meat color of red chromaticity (a*), yellowness index (b*), and luminance (L*) were measured following the procedures described by Shackelford et al. (19).

### 2.8 Statistical analysis

The experimental data were analyzed using MIXED models by SAS 9.4 (SAS Institute Inc., Cary, NC, United States). A randomized complete block design with repeated measures was employed for data analysis. The *Rosa roxburghii* Tratt residue levels (0, 10, 20, and 30%) were designated as fixed effects, with sheep as the random effect. Tukey’s method was adopted to determine differences among mean values, with statistical significance defined as *p* < 0.05. Three sheep data in control, treatment 2, and treatment 3 were excluded from the analysis due to foot injuries, leading to decreased final body weight relative to initial body weight.

## 3 Results

### 3.1 Growth performance and economic benefits

As shown in Table 2, no significant differences were observed in the growth performance indicators (dry matter intake, average daily gain) among the four blocks of Hu sheep. The inclusion of three *Rosa roxburghii* Tratt residue-containing diets for treatment 1, treatment 2, and treatment 3 reduced the feed price relative to control, leading to decreased feed costs as the proportion of *Rosa roxburghii* Tratt residue in the diet increased for treatment 1, treatment 2, and treatment 3. Sheep fed treatment 1 and treatment 2 showed reduced gross profit compared with control and treatment 3. The highest gross profit was observed in treatment 3, surpassing that of control.

**Table 2:** Effects of varying levels of *Rosa roxburghii* Tratt residue on growth performance and economic benefits of Hu sheep.

	Control	Treatment 1	Treatment 2	Treatment 3	SEM	*p*-value
Growth performance						
Initial weight, kg	34.23	34.49	34.40	34.63	0.19	0.55
Dry matter intake, g/d	2086.31	2072.86	2114.67	2160.34	101.04	0.93
Final weight, kg	42.38	42.01	41.64	42.47	0.56	0.72
ANG, kg	8.15	7.52	7.16	7.71	0.55	0.65
Average daily gain, g/d	232.87	214.81	204.49	220.39	15.67	0.65
F/G	9.01	9.71	10.36	9.51	0.73	0.65
Economic benefit ^a^ (CNY/sheep)						
Feed price, CNY/Kg	3.40	3.25	3.10	2.95		
Feed cost	248.27	235.79	229.44	223.06		
Weight gain income	260.80	240.64	231.68	250.88		
Gross profit	12.53	4.85	2.24	27.82		

^a^The price of live Hu sheep is 32 CNY/kg during the trial. Control: basal diet + 0% *Rosa roxburghii* Tratt residue; Treatment 1: basal diet + 10% *Rosa roxburghii* Tratt residue; Treatment 2: basal diet + 20% *Rosa roxburghii* Tratt residue; Treatment 3: basal diet + 30% *Rosa roxburghii* Tratt residue.

### 3.2 Plasma metabolites

Table 3 presents the effects of varying levels of *Rosa roxburghii* Tratt residue on plasma metabolites of Hu sheep. Compared to control, the plasma albumin content was lower in treatment 1, treatment 2, and treatment 3 (*p* < 0.05). Treatment 3 resulted in decreased plasma Crea compared to control and treatment 1 (*p* < 0.05). Plasma high-density lipoprotein content was higher in treatment 2 and treatment 3 relative to control and treatment 1 (*p* < 0.05). The plasma total cholesterol content of treatment 2 and treatment 3 was significantly higher than that of control (*p* < 0.05). No significant differences were observed in other plasma metabolites among these four groups.

**Table 3:** Effects of varying levels of *Rosa roxburghii* Tratt residue on plasma metabolites of Hu sheep.

Item	Control	Treatment 1	Treatment 2	Treatment 3	SEM	*p*-value
TP, g/L	60.76	60.71	60.69	60.82	0.04	0.17
ALB, g/L	28.27^a^	25.32^b^	24.25^b^	23.83^b^	0.67	<0.01
ALP, U/L	257.79	336.38	284.56	287.72	45.87	0.68
ALT, U/L	12.43	13.26	11.91	11.68	1.03	0.71
AST, U/L	106.99	115.35	95.59	88.85	9.85	0.25
AST/ALT	8.84	9.48	8.44	7.96	0.96	0.72
Crea, μmol/L	51.51^a^	48.67^a^	45.59^ab^	41.73^b^	2.24	0.02
HDL, mmol/L	0.77^b^	0.95^ab^	1.18^a^	1.32^a^	0.13	0.02
TC, mmol/L	1.14^b^	1.47^ab^	1.92^a^	2.12^a^	0.25	0.03
TG, mmol/L	0.33	0.34	0.29	0.34	0.03	0.73
Urea, mmol/L	3.32	3.41	3.43	2.56	0.29	0.11

^a,b^Means in the superscript differs in the same row (*p* < 0.05). Control: basal diet + 0% *Rosa roxburghii* Tratt residue; Treatment 1: basal diet + 10% *Rosa roxburghii* Tratt residue; Treatment 2: basal diet + 20% *Rosa roxburghii* Tratt residue; Treatment 3: basal diet + 30% *Rosa roxburghii* Tratt residue.

### 3.3 Rumen fermentation parameters

Feeding varying levels of *Rosa roxburghii* Tratt residue did not exert a difference in levels of rumen pH and N-NH_3_. The rumen total volatile fatty acids, acetic acid, propionic acid, butyric acid, and methane were also unaffected for Hu sheep consuming the four diets (Table 4).

**Table 4:** Effects of varying levels of *Rosa roxburghii* Tratt residue on rumen fermentation paramenters of Hu sheep.

	Control	Treatment 1	Treatment 2	Treatment 3	SEM	*p*-value
pH	6.72	6.75	6.77	6.73	0.04	0.89
NH_3_-N, mmol/L	11.88	13.44	14.77	14.87	0.96	0.13
Total VFA, mmol/L	146.64	152.30	155.11	159.57	5.47	0.42
Acetic, %	63.29	62.72	63.38	63.32	0.85	0.94
Propionic, %	26.24	26.58	26.81	27.74	0.69	0.47
Butyric, %	8.00	8.06	7.32	6.45	0.54	0.15
Acetic/Propionic	2.45	2.36	2.37	2.29	0.09	0.67
CH_4_, mmol/L	35.99	36.74	37.34	37.38	1.57	0.91

^a,b^Means in the superscript differs in the same row (*p* < 0.05). CH_4_ = 0.45 × Acetic acid-0.275 × Propionic acid + 0.4 × Butyric acid. Control: basal diet + 0% *Rosa roxburghii* Tratt residue; Treatment 1: basal diet + 10% *Rosa roxburghii* Tratt residue; Treatment 2: basal diet + 20% *Rosa roxburghii* Tratt residue; Treatment 3: basal diet + 30% *Rosa roxburghii* Tratt residue.

### 3.4 Slaughter performance and organ index

No significant differences were observed for the slaughter performance and organ index of Hu sheep fed varying levels of *Rosa roxburghii* Tratt residue (Table 5).

**Table 5:** Effects of varying levels of *Rosa roxburghii* Tratt residue on slaughter performance of Hu sheep.

	Control	Treatment 1	Treatment 2	Treatment 3	SEM	*p*-value
Pre-slaughter live weight, kg	37.01	38.32	37.47	38.38	1.33	0.86
Dressed weight, kg	34.75	35.87	34.15	35.40	1.34	0.81
Carcass weight, kg	20.19	19.64	18.72	18.70	0.69	0.36
Carcass percentage, %	51.47	51.20	50.00	49.90	0.63	0.21
Heart weight, g	141.85	144.78	147.85	146.98	4.03	0.73
Liver-gallbladder weight, g	598.37	609.22	572.33	582.82	24.77	0.73
Kidney weight, g	91.10	92.82	94.77	93.02	2.97	0.86
Lung weight, g	884.10	782.15	776.75	768.43	80.61	0.72
Intestines weight, kg	1.40	1.38	1.53	1.48	0.06	0.24
Stomach weight, kg	1.07	1.10	1.05	1.05	0.06	0.91

^a,b^Means in the superscript differs in the same row (*p* < 0.05). Control: basal diet + 0% *Rosa roxburghii* Tratt residue; Treatment 1: basal diet + 10% *Rosa roxburghii* Tratt residue; Treatment 2: basal diet + 20% *Rosa roxburghii* Tratt residue; Treatment 3: basal diet + 30% *Rosa roxburghii* Tratt residue.

### 3.5 Meat quality

As listed in Table 6, compared with control and treatment 1, sheep in treatment 2 and treatment 3 showed decreased water holding capacity (*p* < 0.05). Correspondingly, the shear force of treatment 2 and treatment 3 was increased compared to that of control and treatment 1 (*p* < 0.05). There were no significant differences in pH, red chromaticity (a*), yellowness index (b*), and luminance (L*).

**Table 6:** Effects of varying levels of *Rosa roxburghii* Tratt residue on meat quality of Hu sheep.

	Control	Treatment 1	Treatment 2	Treatment 3	SEM	*p*-value
pH_45 min_	5.80	6.03	5.81	5.85	0.13	0.54
pH_24 h_	5.63	5.64	5.57	5.62	0.05	0.79
Water holding capacity (%)	15.01^a^	13.68^a^	7.93^b^	7.60^b^	1.04	<0.01
Shear force (N)	45.59^b^	41.84^b^	57.79^a^	57.49^a^	1.58	<0.01
Red chromaticity (a*)	11.77	13.65	13.07	13.39	0.77	0.34
Yellowness index (b*)	11.13	12.35	12.11	12.06	0.51	0.36
Luminance (L*)	34.08	34.54	34.79	34.61	1.15	0.98

^a,b^Means in the superscript differs in the same row (*p* < 0.05). Control: basal diet + 0% *Rosa roxburghii* Tratt residue; Treatment 1: basal diet + 10% *Rosa roxburghii* Tratt residue; Treatment 2: basal diet + 20% *Rosa roxburghii* Tratt residue; Treatment 3: basal diet + 30% *Rosa roxburghii* Tratt residue.

## 4 Discussion

The livestock industry faces challenges such as rising roughage prices, insufficient supply, and high transportation costs (20). Leveraging fruit residues as alternative feed sources could offer a promising solution. Fruit residues are rich in nutrients, including vitamins, minerals, polyphenols, and dietary fiber (21). Previous studies (22, 23) have shown that they can reduce feed costs. However, while many fruit residues have been explored as substitutes in ruminant production, limited information exists regarding the utilization of *Rosa roxburghii* Tratt residue, a locally unique fruit residue primarily produced in Guizhou province, China. Therefore, this study seeks to assess the feasibility of *Rosa roxburghii* Tratt residue as an alternative roughage for Hu sheep.

### 4.1 Effects of varying levels of *Rosa roxburghii* Tratt residue on growth performance and economic benefits of Hu sheep

The present study revealed that feeding *Rosa roxburghii* Tratt residue had no statistically adverse effects on growth parameters, including dry matter intake, average daily gain, and feed-to-gain ratio. This suggests that *Rosa roxburghii* Tratt residue can serve as a viable roughage option for Hu sheep. However, these findings contradict those of Oduguwa (24), who reported improved feed intake and growth performance in West African dwarf sheep fed pineapple residue. The disparity may stem from differences in fruit residue types. In another study by Hsu et al. (25), it was observed that 15% citrus residue had no effect on average daily gain, but whole citrus residue silage significantly improved feed conversion efficiency compared to dried citrus residue.

Regarding economic benefits, the net gross profit increased gradually with higher levels of *Rosa roxburghii* Tratt residue, peaking at a 30% *Rosa roxburghii* Tratt residue inclusion level. This suggests that *Rosa roxburghii* Tratt residue utilization can reduce feed costs and enhance economic returns. However, it was unexpected to find lower gross profits at 10 and 20% *Rosa roxburghii* Tratt residue levels, possibly due to decreased average daily gain and increased feed-to-gain ratio. We observed that the lowest gross profit for the treatment 2 diet compared to control would remain relatively stable only if the live sheep price decreased by 20.69 CNY/kg, representing a 35.34% reduction. However, such a significant price drop is unlikely to take place. Therefore, in practice, there may be an animal threshold price for substituting low-price fruit residues while considering economic returns. The economic viability of low-price fruit residues will be significantly enhanced with increased live animal prices.

### 4.2 Effects of varying levels of *Rosa roxburghii* Tratt residue on plasma metabolites of Hu sheep

Plasma metabolites are vital indicators of animal health and metabolic activity *in vivo* (26). The study results revealed that, in comparison to the control group, *Rosa roxburghii* Tratt residue treatment significantly reduced plasma albumin levels in treatment 1, treatment 2, and treatment 3 with increasing dosage. Nonetheless, it’s worth noting that all four groups remained within the normal range, indicating minimal detrimental effects on protein metabolism. Plasma Crea, a by-product of protein metabolism, serves as a marker of protein efficiency (27). Typically, a higher blood Crea level correlates with lower protein utilization. Interestingly, in this study, plasma Crea concentration was lower in treatment 3 compared to the control and treatment 1 groups, suggesting that incorporating a high level of R*osa roxburghii* Tratt residue may enhance plasma protein utilization efficiency in Hu sheep (28). The authors assume that tannin might be the specific compound within the *Rosa roxburghii* extract which reduces the protein utilization, because the phenolic hydroxyl groups in tannins could form the insoluble complexes with proteins which cannot be used by animals (29). In the review of Wang et al. Who determined the tannin level is 1.6 g (calculated per 100 g edible part) (7). Triglyceride, a measure of lipid metabolism, primarily consists of low-density lipoprotein and high-density lipoprotein. High-density lipoprotein has the capacity to transport cholesterol out of cells, thereby reducing blood cholesterol levels (30). Zeweil et al. (31) reported that rabbits showed increased blood high-density lipoprotein content when pomegranate was added to their diet. The heightened plasma high-density lipoprotein levels in treatment 2 and treatment 3 groups in our study may imply that *Rosa roxburghii* Tratt residue is beneficial for lipid metabolism. According to the study (32), the dry matter (DM) of pomegranate pomace was 33.02%, which is higher than *Rosa roxburghii* Tratt residue (31.50%); the crude protein (CP) of pomegranate pomace was 9.2%, which is lower than *Rosa roxburghii* Tratt residue (11.99%); the neutral detergent fiber (NDF) and acid detergent fiber (ADF) respectively were 35.35 and 30.61%, which is lower than *Rosa roxburghii* Tratt residue (53.71 and 47.22%). No significant differences were observed for other plasma metabolites.

Effects of varying levels of *Rosa roxburghii* Tratt residue on rumen fermentation parameters of Hu sheep.

In this study, no significant differences were found for rumen fermentation parameters among the four groups of sheep. These results indicate that *Rosa roxburghii* Tratt residue inclusion had no adverse impact on rumen fermentation in Hu sheep. Considering the significance of rumen fermentation parameters, it can be concluded that *Rosa roxburghii* Tratt residue is safe for use in ruminant production. However, the limited microbial diversity in the rumen observed in this study warrants further investigation in future research.

### 4.3 Effects of varying levels of *Rosa roxburghii* Tratt residue on slaughter performance and organ index of Hu sheep

Slaughter performance is a crucial measure of livestock productivity in animal feeding (33–35), typically associated with economic benefits. In this study, the carcass percentage slightly decreased with the increased *Rosa roxburghii* Tratt residue level. This result may be attributed to the RRT seed, which constitutes 39.60% of the *Rosa roxburghii* Tratt residue, being too hard to be digested and absorbed by Hu sheep, thus restricting nutrient utilization. Therefore, finding effective methods to better utilize *Rosa roxburghii* Tratt seed in future research is necessary. The organ index remained unaffected by *Rosa roxburghii* Tratt residue treatment.

### 4.4 Effects of varying levels of *Rosa roxburghii* Tratt residue on meat quality of Hu sheep

Meat quality is a general evaluation based on different kinds of meat quality traits, which greatly influence the choice of consumers (36). Meat sensory quality indicators, including pH, water holding capacity, shear force, and color, are critical factors influencing consumer preference (37). Shear force is a crucial indicator of meat tenderness and is negatively correlated with the meat’s water holding capacity. Meat becomes tougher with increased shear force or decreased water holding capacity. In our study, the increased shear force and decreased water holding capacity in treatment 2 and treatment 3 compared to control and treatment 1 may suggest that *Rosa roxburghii* Tratt residue can influence meat tenderness. Further investigation is needed to determine the reasons for this. No other significant differences were observed in meat sensory quality.

Since *Rosa roxburghii* Tratt residue is a local and novel feed resource, according to the authors’ knowledge, it has not been reported using as a feed resource till now, therefore, we take the proportion of other fruit residues added into ruminant feed as the reference adding level in the current study. Taasoli and Kafilzadeh (38) respectively provided 30% ensiled apple residue and 20% dried apple residue, on dry matter basis, to Sanjabi male lambs; Gowda et al. (39) fed a total mixed ration containing 62% silage pineapple fruit residue and 48% concentrate mixture (dry matter basis) to sheep; Tayengwa and Mapiye (40) summarized the effect of dietary supplements citrus and winery wastes (grape pomace) for ruminant animal, the amount of citrus residue added is 25 ~ 400 g/kg for lambs, 90 ~ 180 g/kg for cows, 50 ~ 200 g/kg for calves, and the amount of grape pomace added is 762 g/kg for lambs, 300 g/kg for wethers, 100 g/kg for steers.

## 5 Conclusion

Overall, the inclusion of up to 30% *Rosa roxburghii* Tratt residue in the diet had no effect on growth performance and could save feed costs for Hu sheep. *Rosa roxburghii* Tratt residue is beneficial for improving plasma protein efficiency and enhancing lipid metabolism. The use of *Rosa roxburghii* Tratt residue does not affect rumen fermentation parameters. Feeding *Rosa roxburghii* Tratt residue has no effect on slaughter performance and organ index, and it resulted in a limited effect on meat quality. When considering a low-price diet, *Rosa roxburghii* Tratt residue is a good choice, but its seed requires further processing for better utilization, and there might be a threshold level in practice to ensure economic income. Therefore, based on the available results and taking into account the cost-effectiveness of supplemental *Rosa roxburghii* Tratt residue, we recommend an optimal inclusion level of 30% for *Rosa roxburghii* Tratt residue.

## Data availability statement

The original contributions presented in the study are included in the article/supplementary material, further inquiries can be directed to the corresponding author.

## Ethics statement

The animal study was approved by Animal Ethics Committee of Guizhou University. The study was conducted in accordance with the local legislation and institutional requirements.

## Author contributions

HL: Conceptualization, Writing – original draft. XS: Writing – original draft, Investigation, Data curation. WW: Writing – review & editing, Supervision, Project administration. CZ: Writing – review & editing, Validation, Funding acquisition.
